# Impact of Hydraulic Resistance on Spatiotemporal Characteristics of Initial Six Steps When Sprinting Under Varying Loads

**DOI:** 10.3390/jfmk9040263

**Published:** 2024-12-08

**Authors:** Matic Sašek, Žiga Leban, Sara Kranjc, Nejc Šarabon

**Affiliations:** 1 Faculty of Health Sciences, University of Primorska, 6310 Izola, Slovenia; matic.sasek@fvz.upr.si (M.S.);; 2 Andrej Marušič Institute, University of Primorska, 6000 Koper, Slovenia; 3 Laboratory for Motor Control and Motor Behavior, S2P, Science to Practice, Ltd., 1000 Ljubljana, Slovenia; 4 Ludwig Boltzmann Institute for Rehabilitation Research, 1010 Vienna, Austria

**Keywords:** resisted sprints, hydraulic resistance, spatiotemporal characteristics, sprint acceleration, speed training

## Abstract

**Background:** Evaluations of the usability of hydraulic resistance for resisted sprint-training purposes remains rare. Thus, this study compared step-by-step changes in spatiotemporal characteristics during the first 10 m of sprints with varying hydraulic resistance loads. **Methods:** Fourteen male athletes performed 20 m sprints under minimal (10 N, considered as normal sprint), moderate (100 N), and heavy (150 N) hydraulic resistance loads. Split times at 10 m, contact time (CT), step length (SL), flight time, and step speed (SS) from the first to the sixth step were measured. A two-way repeated measures ANOVA (load × step) and a one-way ANOVA (load) with post hoc comparisons were used to assess the effects on spatiotemporal characteristics and split times, respectively. **Results:** Under higher loads, the 10 m times were significantly longer (η^2^ = 0.79). The CT, SL, and SS varied significantly from step to step within all loads (η^2^ = 0.45, 0.41, and 0.54, respectively). The CT, SL, and SS of the first, fourth, fifth, and sixth steps of normal sprint differed significantly from most steps under moderate and heavy load (Cohen’s *d* = −3.09 to 5.39). In contrast, the smallest differences were observed between the second and third step of normal sprint and second to sixth steps under heavy load (Cohen’s *d* = −0.67 to 1.32, and −0.71 to 1.38, respectively). **Conclusions:** At the same load settings, a hydraulic resistance device induces changes in step characteristics comparable with those of other motorized devices and is therefore a viable option for resisted sprint training. If the goal of the training is to replicate the steps of the initial sprint acceleration phase, ~150 N of hydraulic resistance would be optimal.

## 1. Introduction

Resisted sprinting is a unique form of training that has received a lot of attention from the research community over the last decade [1,2]. Interestingly, it has been shown that sprint training with loads between 10 and more than 50% of the maximal sprint velocity decrease is one of the most efficient stimuli to improve sprint acceleration performance [3,4]. These observations can be attributed to several factors, including the high production of horizontal ground reaction forces when sprinting against an additional external resistance [5,6] and the replication of body positions [7,8], step length (SL), contact time (CT), step speed (SS), and flight time (FT) that resemble the acceleration phase of a normal sprint [9,10]. These characteristics make resisted sprinting a highly specific training modality [11,12]. To date, various equipment and resistance levels have been used for practical purposes and tested in research [11,13].

Uphill sprints [14], sleds [11,15], and parachutes [16,17] represent the basic tools for resisted sprinting. The effects of training with such equipment on sprint performance have been combined in meta-analyses. Hamad et al. [14] found similar improvements between uphill sprints and resisted sprints, while Petrakos et al. [1] and Aldrich et al. [4] found positive improvements when using sleds and parachutes. These observations confirm that numerous positive effects can be achieved with basic equipment. However, precise individualization and optimization of the training and loads when sprinting uphill, with sleds, or with parachutes is a challenge [18]. Resisted sprint training can be easily optimized with motorized devices and has been presented in the literature in recent years [18,19,20,21,22,23,24,25,26]. Motorized devices allow different levels of resistance to be provided with high precision and several studies have shown that the step kinetics and kinematics in the desired acceleration phase of normal sprinting can be replicated in the later phases of sprinting with lower or higher motorized resistance [5,22,27,28].

While the effects of friction (i.e., sleds), air resistance (i.e., parachutes), or motorized resistance on CT, SL, FT and SS are well documented, the effects of hydraulic resistance in this context have been little researched. The hydraulic system as a resistance device (hereafter referred to as the hydraulic resistance device) provides consistent and precise resistance in a range of 10 to 150 N and could provide a more cost-effective solution for training [29]. Detailed step-by-step changes in spatiotemporal step characteristics due to increasing hydraulic resistance are scarce, raising questions about the usability and specificity of hydraulic resistance devices for resisted sprint training and the replication of normal sprint acceleration kinematics.

To fill these gaps, this study aimed to compare the steps during the first 10 m of sprints with different hydraulic resistance loads. We hypothesize that significant step-by-step changes in spatiotemporal characteristics will occur at different resistance loads and that there will be significant differences in these variables between loads. We also expect that the spatiotemporal characteristics will be similar between the initial sprint steps with minimal resistance and the later sprint steps with higher hydraulic resistance.

## 2. Materials and Methods

A within-subject, cross-sectional study design was employed. During a single session, participants performed a series of 20 m sprints under minimal, moderate and heavy hydraulic resistance loads. Split times for the 10 and 20 m were recorded, along with step-by-step spatiotemporal characteristics (CS, SL, FT, and SS) over the first 10 m of the sprint. All variables were later compared across different loads and individual steps.

### 2.1. Participants

Fourteen male athletes from track and field, soccer, triathlon, and futsal (mean ± standard deviation age 24.9 ± 6.5 years, body mass 78.6 ± 6.9 kg, and body height 1.81 ± 6.8 m, 9.5 ± 3.5 years of training, 6.1 ± 1.1 training sessions per week) ranging from professional to amateur levels were recruited for the study. All participants had prior experience with resisted sprints. They were instructed to refrain from any lower-body strength or speed training within 48 h before the testing procedure. Informed consent, including a description of the procedures and potential risks was obtained and signed by participants, or by their parents/legal guardians if the participants were under 18 years of age. All participants were healthy and had no musculoskeletal injuries of the back or lower limbs in the past 9 months that could affect the study’s results. The study was conducted in accordance with the Declaration of Helsinki and was approved by the Medical Ethics Committee under grant number 0120-690/2017/8.

### 2.2. Procedures

**Figure 1:**
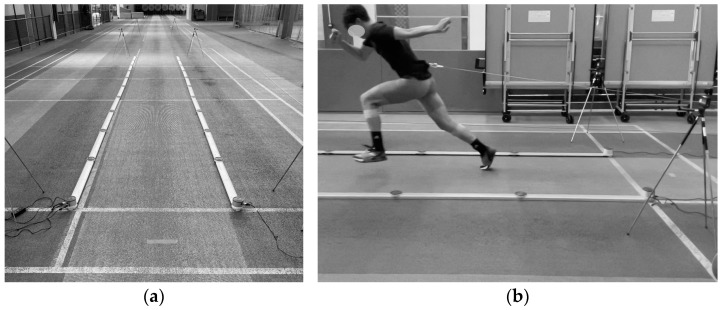
The setup for assessment of step spatiotemporal characteristics and split times at 10 and 20 m. (**a**) Timing gates and optical measurement system position on the track; (**b**) athlete while sprinting (during the push-off phase of the second step) under moderate hydraulic resistance load.

The testing procedure took place indoors on a synthetic surface (i.e., rubber track and field floor). All experiments were performed in flat-soled running shoes. A standardized warm-up routine included low-to-moderate tempo running, two sets of eight dynamic stretching and strengthening exercises for the lower limbs, 20 m running drills (such as skipping, high knees, and hopping), self-preferred warm-up activities, followed by one submaximal 20 m sprint and one submaximal 20 m resisted sprint with 60 Newtons (N) of resistance. Subsequently, participants performed two consecutive maximal 20 m resisted sprints at each load. Minimal (10 N; note that such a load is considered here as a normal sprint condition [30]), moderate (100 N) and heavy (150 N) hydraulic resistance loads were applied in a randomized order. In accordance with previous studies with a similar design, each sprint was followed by a recovery period of at least 5 min [18]. Resistance was provided by a hydraulic device specifically designed for sprint training and testing, which allows for isotonic adjustable resistance [29]. The device was positioned approximately 4 m behind the start line and connected to participants via a cord attached to a belt (Figure 1b). Sprints were initiated from a two-point standing start at the participant’s initiation.

### 2.3. Data Acquisition and Equipment

Single-beam timing gates (Brower Timing Systems, Draper, UT, USA) with a sampling frequency of 0.01 s were used to measure 10 m split time. The sensors were mounted at hip height and positioned at the start (0.5 m from the participant’s front foot), as well as at the 10 m distance [31]. Since the actual sprint start was initiated before the first set of timing gates were triggered (i.e., a flying start), 0.5 s were added to the split times for all resistance conditions [32].

The spatiotemporal characteristics (SL, CT, FT, and SS) over the first 10 m were measured using a validated optical measurement system (OptojumpNext, Microgate, Bolzano, Italy) [33], with data collected at a frequency of 1000 Hz via Optojump software (version 1.12.21.0, Microgate, Bolzano, Italy). The system was positioned at floor level within the 10 m track zone, which began 0.5 m in front of the participant’s front foot (Figure 1a). Since the sprint start occurred outside of the 10 m measurement zone, the initial (i.e., push-off) step was excluded from further analysis.

### 2.4. Statistical Analysis

Statistical analyses were performed using GraphPad Prism version 9.0.2 (GraphPad Software, Inc., San Diego, CA, USA). All results are presented as mean ± standard deviation. The normality of the data distribution was assessed using the Shapiro–Wilk test. To evaluate the effects of step number and load on the spatiotemporal characteristics, a 6 (step number) × 3 (load) repeated measures analysis of variance (ANOVA) was conducted. In case of significant effect, post hoc analyses were performed with Holm–Bonferroni adjustments to identify differences between consecutive steps and loads. When the assumption of sphericity was violated, Greenhouse–Geisser adjustments of the *p*-values were reported. Additionally, a one-way ANOVA with load as the factor was used to assess differences in split times. Eta squared (η^2^) was calculated for the ANOVAs and considered as having small (0.01 < η^2^ < 0.06), medium (0.06 < η^2^ < 0.14), and large (η^2^ < 0.14) effect size. A Cohen’s *d* was used to interpret differences between steps and loads and was classified as trivial (<0.2), small (0.2–0.6), moderate (0.6–1.2), large (1.2–2.0), very large (2.0–4.0), or nearly perfect (>4.0) effect size [34]. The level of statistical significance was set at *p* < 0.05.

## 3. Results

All variables were normally distributed. The lowest number of steps performed over 10 m distance was six, therefore, only these steps were included in the comparisons. Descriptive statistics for 10 m split time, FT, CT, SL, and SS are presented in Table 1.

**Figure 2:**
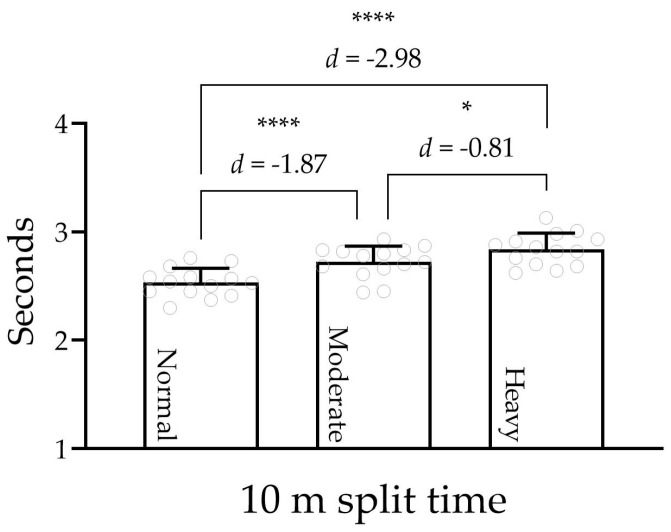
Differences in 10 m split times between normal sprint, moderate load, and heavy load. Please note that the gray circles represent individual split times. * *p* < 0.05; **** *p* < 0.0001.

The one-way ANOVA revealed a significant effect of load on 10 m (F = 48, *p* < 0.0001, η^2^ = 0.79). Post hoc pairwise comparisons indicated that the 10 m and 20 m split times were significantly longer under moderate and heavy load compared to normal sprints (Figure 2). Specifically, under heavy load, the 10 m split time increased by 15.2% (range = 4.8–24.6%). Under moderate load, the increase was 9.5% (range = 1.8–16.8%).

**Figure 3:**
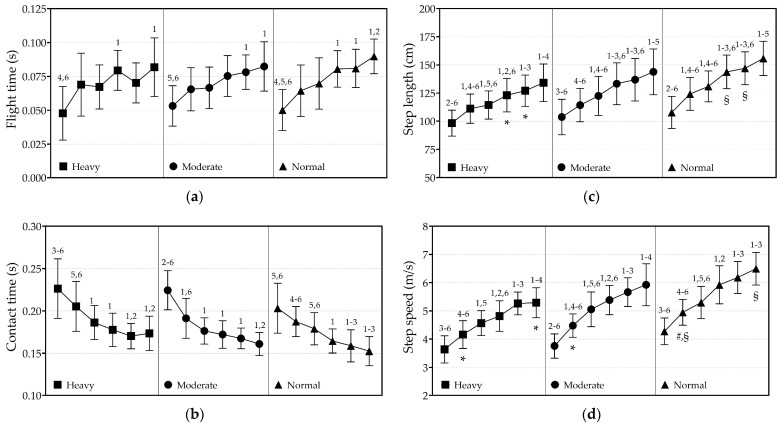
The characteristics of the first, second, third, fourth, fifth, and sixth step under normal sprint, moderate, and heavy hydraulic resistance load conditions. Step-by-step changes within each condition and differences in the same steps between conditions for: (**a**) flight time; (**b**) contact time; (**c**) step length; and (**d**) step speed. ^1^ significantly different as the first step; ^2^ significantly different as the second step; ^3^ significantly different as the third step; ^4^ significantly different as the fourth step; ^5^ significantly different as the fifth step; ^6^ significantly different as the sixth step; * significantly different as the same step of normal sprint; ^#^ significantly different as the same step of moderate load; ^§^ significantly different as the same step of heavy load.

Step-by-step changes in FT, CT, SL, and SS across the three different loads are illustrated in Figure 3. The differences between the steps during normal sprint and those at moderate and heavy load are detailed in Table 2 and Table 3. The two-way ANOVA revealed a significant effect of step number on FT (F = 29.1, *p* < 0.0001, η^2^ = 0.31). Across all loads, the FT of the first step was significantly shorter than that of the sixth step (Cohen’s *d* range = 1.53–2.87). Additionally, the FT of the first step of normal sprint was significantly shorter than the FT of the fourth step under heavy load, as well as the FT of the fourth, fifth, and sixth step under moderate load (Table 2).

No significant interaction was observed for CT; however, significant main effects of load (F = 12.1, *p* = 0.0014, η^2^ = 0.05) and step number (F = 56.6, *p* < 0.0001, η^2^ = 0.45) were identified. The CT of the same steps between loads were not significantly different, whereas, within all loads, the CT decreased as the step number increased (Cohen’s *d* range = −3.24 to −0.67). Moreover, the CT of the first step of normal sprint was significantly longer than the fourth and fifth step under moderate and heavy load. Additionally, the CT of the second step of normal sprint was significantly longer than of the fifth and sixth step under moderate load (Table 2).

A significant interaction effect was found for SL (F(4.4, 56.7) = 4.9, *p =* 0.0014; η^2^ = 0.01). As shown in Figure 3, SL increased significantly from the first to the sixth step, regardless of the applied load (Cohen’s *d* range = 1.19 to 5.5). The fourth and fifth step of normal sprint were significantly longer than the same steps under heavy load (mean difference = 20.71 cm, 95% CI = 1.01 to 40.42 cm, *p =* 0.032; mean difference = 19.93 cm, 95% CI = 0.69 to 39.17 cm, *p =* 0.037, respectively). Additionally, the fourth, fifth and sixth step of normal sprint were significantly longer than most steps under moderate and heavy load. The first step of normal sprint was significantly longer than the sixth step under heavy load, as well as the fourth, fifth, and sixth step under moderate load. Furthermore, the second and third step of normal sprint were significantly longer than the first step under moderate and heavy load (Table 3).

Significant effects on SS were observed for step number (F(2.6, 33.4) = 114.2, *p* < 0.0001; η^2^ = 0.54) and load (F(1.6, 20.1) = 19.6, *p* < 0.0001; η^2^ = 0.15). Pairwise comparisons revealed an increase in SS with step number across all loads (Cohen’s *d* range = 0.85 to 5.5). The fourth, fifth, and sixth steps of the normal sprint were significantly faster than most steps under moderate and heavy load. The first step of normal sprint was slower than the fifth and sixth step under heavy load, as well as the fourth, fifth, and sixth step under moderate load. Furthermore, the second step of normal sprint was significantly faster than the first and second step under moderate and heavy load. Similarly, the third step of normal sprint was significantly faster than the first step under moderate and heavy load.

## 4. Discussion

The results of this study demonstrated that adding hydraulic resistance during 10 m sprints significantly impacts performance. During normal sprints, moderate load, and heavy load sprints, both SL and SS increased, while CT decreased progressively within the first 10 m. However, the trajectory of these changes differs slightly between conditions. With greater load, longer CT and reduced SS and SL were observed. Consequently, the step characteristics of the second and third step of normal sprints were similar to the second to sixth steps of the heavy load sprint. This suggests that the sprint technique akin to the initial acceleration of normal sprint can be replicated across multiple steps when using heavy hydraulic resistance. These findings align with previous evidence on sled and motorized resisted sprints [27,28,35], further confirming the hydraulic resistance device as a specific training modality for sprint acceleration enhancement.

The observed increase in 10 m split time due to increased hydraulic resistance is not surprising. Chang et al. [27] reported that in national-level athletes, 10 m sprint times increased by 33% when 140 N of resistance was applied. For comparison, in our study, applying 100 N (i.e., moderate load) of hydraulic resistance led to a 9.5% increase in 10 m sprint times, while 150 N (i.e., heavy load) resulted in a 15.2% increase in 10 m sprint times. We infer that smaller proportional increases in split times regardless of similar resistance forces provided by devices are likely due to methodological factors rather than the nature of hydraulic resistance itself. In our study, changes in split times due to additional load were compared against 10 N resistance, not against unloaded sprints, which could result in a proportionally smaller increase in 10 m split time. Additionally, Chang et al. [27] employed kinematic assessments for timing, which capture sprint starts more precisely. On the other hand, we used timing gates and estimated that the time of start (i.e., the body’s horizontal movement before the first timing gate was triggered) was consistent across loads. Thus, variability in start times between loads was not accounted for in our study. As the sprint start with heavy load was likely slower, it could potentially lead to a general underestimation of the difference in split times between moderate load, heavy load, and normal sprint conditions (i.e., 10 N).

In this study, it was assumed that the spatiotemporal step characteristics of steps under 10 N of resistance would behave similarly to those in unloaded sprints, though this is not ideal. However, this assumption is reasonably supported, as the characteristics of the six steps of normal sprint are comparable to unloaded sprints reported in the literature. Changes in FT, CT, and SL from the first to the sixth steps of normal sprints (see Table 1) are similar to unloaded sprint data from Chang et al. [27] and van den Tillaar and Gamble [35], who reported changes in FT from 0.056 to 0.096 s and 0.050 to 0.084 s, CT from 0.191 to 0.132 s and 0.188 to 0.125 s, and SL from 108 to 160 cm and 107 to 163 cm, respectively. Furthermore, SL and CT during heavy hydraulic load sprints are comparable to the spatiotemporal characteristics of motorized-resisted sprints at 140 N [27] and 152 ± 10 N (20% of body mass) [28] (SL: 98.3–134.1 cm vs. 89–131 cm vs. 95–138 cm; CT: 0.226–0.173 s vs. 0.195–0.143 s vs. 0.227–0.150 s). These similarities between studies suggest that hydraulic resistance devices induce changes in step characteristics and sprint technique comparable to other resisted sprint devices under the same load settings. Therefore, the use of a hydraulic resistance device could be suitable for research and training purposes.

In general, resisted sprints should replicate the sprinting technique akin to the acceleration phase of unloaded sprints [2]. However, it remains unclear whether, and to what extent, the initial steps in the acceleration phase of a sprint can be reflected across the steps when using hydraulic resistance. Similarly, as previously noted by van den Tillaar [28] for motorized resistance, we observed that the spatiotemporal characteristics change progressively from the first to the sixth step, regardless of the applied hydraulic load (i.e., moderate or heavy). The trajectory of these changes seems to be specific for each load, and the characteristics of the same steps under moderate and heavy loads were different from those under normal sprint conditions. Interestingly, the initial steps of a normal sprint were similar to the later steps with moderate and heavy loads. No significant differences in SL and CT were found between the second and third steps of a normal sprint and the second to sixth step of heavy load. By contrast, the first, fourth, fifth, and sixth steps of a normal sprint were significantly shorter or longer than the second and third step.

Over the first 10 m of a sprint, a heavy hydraulic load replicates the sprinting technique used during the early acceleration phase. By performing sprints with such a load, the neuromuscular system is exposed to specific biomechanical stimuli over more steps compared to normal sprints. Thus, when the target is to improve a particular phase of sprint acceleration (for instance, the second and third step), hydraulic-resisted sprints could be a more economical training modality than short sprints, which are traditionally employed to improve sprint acceleration [12]. This is because fewer trials are needed to provide the same amount of specific training stimuli (i.e., steps in the early acceleration phase). However, these indications are only theoretical assumptions, and further studies are needed to clarify the effects of hydraulic-resisted sprint training on acceleration performance.

The results of this study also open avenues for future research and application of the hydraulic resistance device in practice. This study focused on spatiotemporal step characteristics where a hydraulic device was used primarily for the application of resistance. Additionally, the device allows the measurement of resistance force and power during sprint, which increases its usefulness for research and training purposes. However, these characteristics of the device should be evaluated in future studies. Furthermore, due to the small sample size, there is a need to investigate the effects of hydraulic resistance on a wider range of participants and loading conditions. Extending this work will deepen our understanding of how resisted sprint training with hydraulic resistance can be optimized to enhance performance across a range of athletes and training goals.

## 5. Conclusions

Hydraulic resistance is a practical and effective option for resisted sprint training. Using moderate and heavy hydraulic resistance during sprinting induces changes in performance and step spatiotemporal characteristics akin to those observed with motorized resistance devices. When coaches aim to expose athletes to stimuli that replicate the early acceleration phase of a sprint over an extended number of steps, short sprints with heavy load (150 N hydraulic resistance force) seem to be the most optimal choice.

## Figures and Tables

**Table 1:** Descriptive statistics for flight time, contact time, step length, step speed, and split times of normal sprint, moderate, and heavy hydraulic resistance loads.

Variable	Step	Normal Sprint	Moderate Load	Heavy Load
Flight time (s)	1st	0.051 ± 0.014	0.053 ± 0.015	0.047 ± 0.019
	2nd	0.064 ± 0.019	0.064 ± 0.016	0.067 ± 0.023
	3rd	0.070 ± 0.018	0.066 ± 0.015	0.065 ± 0.014
	4th	0.080 ± 0.012	0.075 ± 0.016	0.078 ± 0.015
	5th	0.079 ± 0.013	0.076 ± 0.013	0.069 ± 0.015
	6th	0.088 ± 0.012	0.082 ± 0.018	0.081 ± 0.020
Contact time (s)	1st	0.204 ± 0.030	0.223 ± 0.023	0.226 ± 0.035
	2nd	0.188 ± 0.018	0.192 ± 0.024	0.203 ± 0.029
	3rd	0.178 ± 0.018	0.177 ± 0.016	0.185 ± 0.020
	4th	0.164 ± 0.014	0.172 ± 0.016	0.178 ± 0.018
	5th	0.159 ± 0.019	0.165 ± 0.013	0.172 ± 0.015
	6th	0.152 ± 0.016	0.162 ± 0.014	0.173 ± 0.019
Step length (cm)	1st	107.8 ± 14.3	103.7 ± 15.7	98.3 ± 11.5
	2nd	124.2 ± 14.6	114.3 ± 14.8	111.1 ± 13
	3rd	130.9 ± 13.7	122.5 ± 17.4	114.4 ± 12.5
	4th	143.8 ± 15.0	133.3 ± 8.6	123.1 ± 14.8
	5th	147.0 ± 14.6	136.9 ± 18.9	127.1 ± 13.9
	6th	155.8 ± 15.2	143.9 ± 20.4	134.1 ± 16.7
Step speed (m/s)	1st	4.28 ± 0.47	3.76 ± 0.43	3.64 ± 0.48
	2nd	4.95 ± 0.46	4.48 ± 0.41	4.16 ± 0.49
	3rd	5.30 ± 0.57	5.06 ± 0.62	4.57 ± 0.44
	4th	5.93 ± 0.68	5.39 ± 0.52	4.82 ± 0.54
	5th	6.19 ± 0.57	5.67 ± 0.51	5.26 ± 0.40
	6th	6.50 ± 0.58	5.93 ± 0.74	5.29 ± 0.53
10 m split time		2.53 ± 0.13	2.72 ± 0.15	2.84 ± 0.15

**Table 2:** Mean differences (with corresponding Cohen’s *d*) in flight time and contact time for comparisons between normal sprint and heavy load, and between normal sprint and moderate load.

Step		Normal Sprint vs. Heavy Load						Normal Sprint vs. Moderate Load					
		1st	2nd	3rd	4th	5th	6th	1st	2nd	3rd	4th	5th	6th
Flight time (s)	1st	0.002	−0.019	−0.017	**−0.029 ^†^**	−0.020	−0.032 *	−0.002	−0.013	−0.015	−0.024	−0.025 *	−0.031 *
		*d = 0.18*	*d = −0.79*	*d = −0.76*	*d = −1.53*	*d = −1.00*	*d = −1.61*	*d = −0.15*	*d = −0.84*	*d = −0.84*	*d = −1.33*	*d = −1.77*	*d = −1.58*
	2nd	0.017	0.005	−0.003	−0.015	−0.006	−0.017	0.012	0.000	−0.002	−0.011	−0.011	−0.018
		*d = 0.63*	*d = −0.16*	*d = −0.06*	*d = −0.76*	*d = −0.26*	*d = −0.81*	*d = 0.62*	*d = 0.02*	*d = −0.08*	*−0.50*	*d = −0.74*	*d = −0.92*
	3rd	0.022	0.001	0.003	−0.01	0.000	−0.012	0.018	0.006	0.004	−0.005	−0.005	−0.012
		*d = 0.84*	*d = 0.17*	*d = 0.28*	*d = −0.55*	*d = 0.02*	*d = −0.51*	*d = 0.78*	*d = 0.36*	*d = 0.27*	*d = −0.28*	*d = −0.38*	*d = −0.73*
	4th	0.033 *	0.012	0.013	0.001	0.010	−0.001	0.028 *	0.016	0.014	0.005	0.005	−0.002
		*d = 1.45*	*d = 0.68*	*d = 0.82*	*d = 0.14*	*0.73*	*d = −0.08*	*d = 1.67*	*d = 1.10*	*d = 0.89*	*d = 0.36*	*d = 0.40*	*d = −0.11*
	5th	**0.033 ^†^**	0.012	0.014	0.002	0.011	−0.001	**0.028 ^†^**	0.015	0.013	0.004	0.004	−0.003
		*d = 1.71*	*d = 0.81*	*d = 1.07*	*d = 0.05*	*d = 0.87*	*d = −0.13*	*d = 1.80*	*d = 0.99*	*d = 1.09*	*d = 0.34*	*d = 0.38*	*d = −0.17*
	6th	**0.042 ^‡^**	0.021	0.023 *	0.01	**0.020 ^†^**	0.008	**0.036 ^‡^**	0.025	**0.023 ^‡^**	0.014	0.013 *	0.007
		*d = 2.18*	*d = 1.24*	*d = 1.71*	*d = 1.01*	*d = 1.63*	*d = 0.64*	*d = 2.19*	*d = 1.62*	*d = 2.00*	*d = 1.29*	*d = 1.65*	*d = 0.48*
Contact time (s)	1st	−0.023	−0.002	0.017	0.026 *	0.033 *	0.030 *	−0.021	0.012	0.027	0.031	0.036 *	0.042 *
		*d = −0.78*	*d = 0.02*	*d = 0.66*	*d = 1.30*	*d = 1.46*	*d = 1.42*	*d = −1.25*	*d = 0.63*	*d = 1.09*	*d = 1.19*	*d = 1.47*	*d = 1.31*
	2nd	−0.039	−0.018	0.001	0.010	0.017	0.014	**−0.037 ^‡^**	−0.004	0.011	0.015	0.020 *	**0.027 ^‡^**
		*d = −1.23*	*d = −0.67*	*d = 0.11*	*d = 0.55*	*d = 1.02*	*d = 0.87*	*d = −2.09*	*d = −0.30*	*d = 0.70*	*d = 0.98*	*d = 1.57*	*d = 1.74*
	3rd	−0.047 *	−0.026	−0.007	0.001	0.009	0.006	**−0.046 ^†^**	−0.012	0.003	0.007	0.011	0.018
		*d = −1.34*	*d = −0.71*	*d = −0.37*	*d = 0.02*	*d = 0.36*	*d = 0.23*	*d = −1.60*	*d = −0.53*	*d = 0.12*	*d = 0.28*	*d = 0.67*	*d = 0.94*
	4th	**−0.062 ^†^**	−0.041 *	−0.022	−0.013	−0.006	−0.009	**−0.060 ^‡^**	−0.027	−0.012	−0.008	−0.003	0.004
		*d = −1.90*	*d = −1.34*	*d = −0.98*	*d = −0.72*	*d = −0.55*	*d = −0.46*	*d = −2.37*	*d = −1.14*	*d = −0.75*	*d = −0.69*	*d = −0.20*	*d = 0.18*
	5th	**−0.068 ^‡^**	**−0.047 ^†^**	−0.028 *	−0.019	−0.012	−0.015	**−0.066 ^§^**	**−0.032 ^†^**	**−0.018 ^†^**	−0.013	−0.009	−0.002
		*d = −2.15*	*d = −1.47*	*d = −1.47*	*d = −0.86*	*d = −0.87*	*d = −0.73*	*d = −2.85*	*d = −1.66*	*d = −1.58*	*d = −0.68*	*d = −0.39*	*d = −0.13*
	6th	**−0.074 ^‡^**	**−0.053 ^†^**	**−0.034 ^‡^**	−0.025	−0.018	−0.021	**−0.072 ^§^**	**−0.039 ^†^**	−0.024 *	−0.02	−0.015	−0.008
		*d = −2.22*	*d = −1.81*	*d = −2.04*	*d = −1.23*	*d = −1.31*	*d = −1.07*	*d = −3.09*	*d = −1.98*	*d = −1.60*	*d = −1.45*	*d = −1.17*	*d = −0.82*

**Note.** For clarity, Cohen’s *d* is written in italic and significant mean differences in bold. * *p* < 0.05; ^†^ *p* < 0.01; ^‡^ *p* < 0.001; ^§^ *p* < 0.0001.

**Table 3:** Mean differences (with corresponding Cohen’s *d*) in step length and step speed for comparisons between normal sprint and heavy load, and between normal sprint and moderate load.

Step		Normal Sprint vs. Heavy Load						Normal Sprint vs. Moderate Load					
		1st	2nd	3rd	1st	2nd	6th	1st	2nd	3rd	1st	2nd	6th
Step length (cm)	1st	9.50	−3.36	−6.57	−15.29	−19.29 *	−26.36 *	4.07	−6.5	−14.71	**−25.50 ^‡^**	**−29.07 ^‡^**	**−36.07 ^‡^**
		*d = 0.98*	*d = −0.24*	*d = −0.49*	*d = −0.88*	*d = −1.29*	*d = −1.38*	*d = 0.55*	*d = −0.48*	*d = −1.25*	*d = −1.95*	*d = −2.17*	*d = −2.22*
	2nd	**25.93 ^‡^**	13.07	9.86	1.14	−2.86	−9.93	**20.5 ^‡^**	9.93	1.71	−9.07	−12.64	**−19.64 ^†^**
		*d = 2.54*	*d = 1.08*	*d = 0.81*	*d = 0.08*	*d = −0.22*	*d = −0.64*	*d = 2.88*	*d = 1.04*	*d = 0.19*	*d = −0.97*	*d = −1.20*	*d = −1.55*
	3rd	**32.64 ^‡^**	19.79 *	16.57	7.86	3.86	−3.21	**27.21 ^‡^**	16.64	8.43	−2.36	−5.93	−12.93
		*d = 2.99*	*d = 1.38*	*d = 1.20*	*d = 0.47*	*d = 0.26*	*d = −0.17*	*d = 2.92*	*d = 1.21*	*d = 0.68*	*d = −0.19*	*d = −0.46*	*d = −0.80*
	4th	**45.50 ^‡^**	**32.64 ^‡^**	**29.43 ^‡^**	20.71 *	16.71	9.64	**40.07 ^‡^**	**29.5 ^‡^**	**21.29 ^‡^**	10.50	6.93	−0.07
		*d = 4.17*	*d = 2.25*	*d = 2.05*	*d = 1.36*	*d = 1.14*	*d = 0.56*	*d = 4.58*	*d = 2.70*	*d = 2.15*	*d = 1.03*	*d = 0.58*	*d = −0.01*
	5th	**48.71 ^‡^**	**35.86 ^‡^**	**32.64 ^‡^**	23.93 *	19.93 *	12.86	**43.29 ^‡^**	**32.71 ^‡^**	**24.5 ^‡^**	13.71 *	10.14	3.14
		*d = 4.69*	*d = 2.43*	*d = 2.29*	*d = 1.44*	*d = 1.34*	*d = 0.70*	*d = 5.16*	*d = 2.87*	*d = 2.40*	*d = 1.31*	*d = 0.90*	*d = 0.22*
	6th	**57.50 ^‡^**	**44.64 ^‡^**	**41.43 ^‡^**	**32.71 ^‡^**	**28.71 ^‡^**	21.64	**52.07 ^‡^**	**41.5 ^‡^**	**33.29 ^‡^**	**22.5 ^‡^**	**18.93 ^†^**	11.93
		*d = 5.37*	*d = 2.95*	*d = 2.89*	*d = 2.12*	*d = 1.94*	*d = 1.24*	*d = 6.21*	*d = 3.82*	*d = 3.66*	*d = 2.43*	*d = 1.76*	*d = 0.94*
Step speed (m/s)	1st	0.64	0.12	−0.29	−0.54	−0.99 *	−1.01 *	0.52	−0.2	−0.78	−1.11 *	**−1.39 ^‡^**	**−1.65 ^†^**
		*d = 0.92*	*d = 0.16*	*d = −0.42*	*d = −0.70*	*d = −1.44*	*d = −1.45*	*d = 0.80*	*d = −0.31*	*d = −0.95*	*d = −1.51*	*d = −2.02*	*d = −1.69*
	2nd	**1.31 ^†^**	0.79 *	0.38	0.13	−0.32	−0.34	**1.19 ^‡^**	0.47 *	−0.11	−0.44	−0.72	−0.98 *
		*d = 1.81*	*d = 1.32*	*d = 0.57*	*d = 0.19*	*d = −0.52*	*d = −0.49*	*d = 2.05*	*d = 1.52*	*d = −0.14*	*d = −0.76*	*d = −1.15*	*d = −1.40*
	3rd	**1.66 ^‡^**	1.14	0.73	0.48	0.03	0.01	**1.54 ^‡^**	0.82	0.24	−0.09	−0.37	−0.63
		*d = 2.07*	*d = 1.15*	*d = 1.18*	*d = 0.49*	*d = 0.04*	*d = 0.01*	*d = 2.29*	*d = 1.18*	*d = 0.44*	*d = −0.15*	*d = −0.54*	*d = −0.81*
	4th	**2.29 ^‡^**	**1.76 ^†^**	**1.36 ^†^**	1.11	0.66	0.63	**2.17 ^‡^**	**1.45 ^‡^**	0.87	0.54	0.26	0.00
		*d = 2.65*	*d = 1.93*	*d = 1.74*	*d = 1.18*	*d = 0.90*	*d = 0.69*	*d = 3.84*	*d = 2.70*	*d = 1.15*	*d = 1.14*	*d = 0.47*	*d = 0.00*
	5th	**2.55 ^‡^**	**2.02 ^‡^**	**1.62 ^‡^**	1.37 *	0.92	0.89	**2.43 ^‡^**	**1.71 ^‡^**	**1.13 ^‡^**	0.80 *	0.52	0.26
		*d = 3.46*	*d = 2.22*	*d = 2.47*	*d = 1.49*	*d = 1.24*	*d = 1.04*	*d = 3.89*	*d = 2.70*	*d = 2.00*	*d = 1.50*	*d = 0.84*	*d = 0.37*
	6th	**2.86 ^‡^**	**2.34 ^‡^**	**1.93 ^‡^**	**1.68 ^†^**	**1.23 ^†^**	1.21 *	**2.74 ^‡^**	**2.02 ^‡^**	**1.44 ^‡^**	**1.11 ^‡^**	0.83 *	0.57
		*d = 3.56*	*d = 2.46*	*d = 2.99*	*d = 1.77*	*d = 1.63*	*d = 1.31*	*d = 4.81*	*d = 3.70*	*d = 2.43*	*d = 2.39*	*d = 1.44*	*d = 0.94*

**Note.** For clarity, Cohen’s *d* is written in italic, and significant mean differences in bold. * *p* < 0.05; ^†^ *p* < 0.01; ^‡^ *p* < 0.001.

## Data Availability

The data that support the findings of this study are available from the corresponding author (Nejc Šarabon) upon reasonable request.
